# Ultrasound Mediated One-Pot, Three Component Synthesis, Docking and ADME Prediction of Novel 5-Amino-2-(4-chlorophenyl)-7-Substituted Phenyl-8,8a-dihydro-7*H*-(1,3,4)thiadiazolo(3,2-α)pyrimidine-6-carbonitrile Derivatives as Anticancer Agents

**DOI:** 10.3390/molecules21080894

**Published:** 2016-07-29

**Authors:** Shailee V. Tiwari, Julio A. Seijas, M. Pilar Vazquez-Tato, Aniket P. Sarkate, Deepak K. Lokwani, Anna Pratima G. Nikalje

**Affiliations:** 1 Y.B. Chavan College of Pharmacy, Dr. Rafiq Zakaria Campus, Rauza Baug, Aurangabad, Maharashtra 431001, India; shailee2010@gmail.com; 2 Departamento de Química Orgánica, Facultad de Ciencias, Universidad of Santiago De Compostela, Alfonso X el Sabio, Lugo 27002, Spain; julioa.seijas@usc.es (J.A.S.); pilar.vazquez.tato@usc.es (M.P.V.-T.); 3 Department of Chemical Technology, Dr. Babasaheb Ambedkar Marathwada University, Aurangabad, Maharashtra 431004, India; aniketpharma1@gmail.com (A.P.S.); dklokwani@gmail.com (D.K.L.)

**Keywords:** 1,3,4-thiadiazolo(3,2-α)pyrimidine, ultrasound-promoted synthesis, ADME, docking

## Abstract

Herein, we report an environmentally friendly, rapid, and convenient one-pot ultrasound-promoted synthesis of 5-amino-2-(4-chlorophenyl)-7-substituted phenyl-8,8a-dihydro-7*H*-(1,3,4)thiadiazolo(3,2-α)pyrimidine-6-carbonitrile derivatives. The in-vitro anticancer activities of these compounds were evaluated against four human tumor cell lines. Among all the synthesized derivatives, compound **4i**, which has substituent 3-hydroxy-4-methoxyphenyl is found to have the highest GI_50_ value of 32.7 μM, 55.3 μM, 34.3 μM, 28.9 μM for MCF-7, K562, HeLa and PC-3 cancer cell lines respectively. A docking study of the newly synthesized compounds were performed, and the results showed good binding mode in the active site of thymidylate synthase enzyme. ADME properties of synthesized compounds were also studied and showed good drug like properties.

## 1. Introduction

Cancer is a disease in which cells grow and proliferate in an uncontrolled manner. Cancer disease evokes a high level of mortality regardless of recent advances in the development of clinically authorized anticancer agents [1]. On the basis of source and action mechanisms, the anticancer drugs are classified as alkylating agents, antimetabolites, natural products, hormones and antagonistic, miscellaneous agents. Many scientists are intensively engaged in the development of new anticancer active agents that reveal a selective cytotoxicity for cancer cells over normal cells which is undoubtedly needed to treat the severe cancer disease more efficiently and is also less toxic, since many of the marketed anticancer drugs are toxic in nature [2,3,4].

Folate metabolism is considered as an important target for the development of new anticancer agents due to its role in the biosynthesis of nucleic acid precursors [5,6]. The inhibition of folate dependent enzymes such as thymidylate synthase (TS), which catalyzes the reductive methylation of deoxyuridylate (dUMP) to thymidylate (dTMP) has also been recognized as an interesting target for drug discovery [7,8]. Inhibition of this enzyme leads to thymineless state, rendering cells incapable of undergoing accurate DNA replication, ultimately resulting in cell death. Classical, antifolate inhibitors of thymidylate synthase often suffer from a number of potential disadvantages when used as anticancer agents. These include impaired uptake due to an alteration of the active transport system required for cellular uptake, as well as the formation of long acting, non-effluxing polyglutamates via folypolyglutamate synthetase, which are responsible for toxicity to normal cells. Nonclassical antifolates such as nolatrexed (AG337), AG331, pyrimethamine, trimethoprim (TMP), piritrexim (PTX) and trimetrexate (TMQ) do not require folate transport systems but enter cells via passive diffusion. Recent advances in non-classical antifolates have been documented in the literature [9,10,11]. To overcome some of the disadvantages of classical thymidylate synthase inhibitors, our team has tried to synthesize novel 5-amino-2-(4-chlorophenyl)-7-substituted phenyl-8,8a-dihydro-7*H*-(1,3,4)thiadiazolo(3,2-α)pyrimidine-6-carbonitrile derivatives.

**Figure 1:**
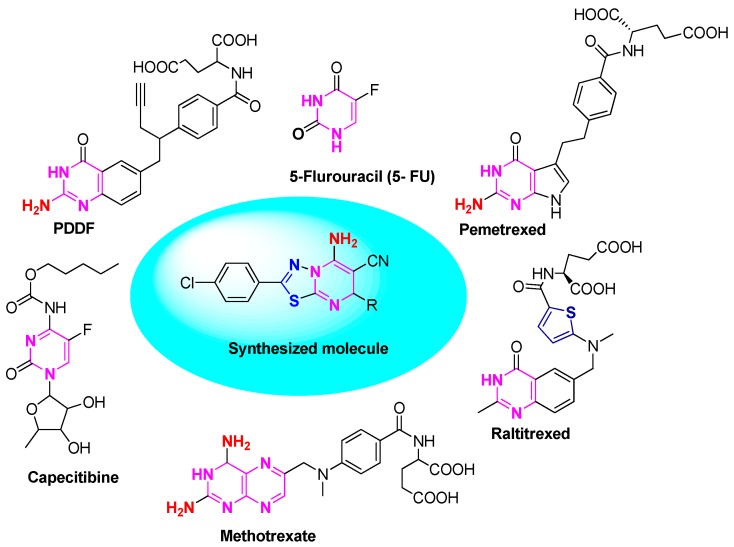
Designing protocol for targeted molecule.

Pyrimidine and its derivatives have been recognized as important heterocyclic compounds due to their variety of chemical and biological significance to medicinal chemistry [12,13,14,15]. Hybridization of two different bioactive molecules with complementary pharmacophoric functions often showed synergistic effects [16,17]. During recent years, there have been intense investigations of fused thiadiazole and pyrimidine systems. Literature survey revealed that (1,3,4)thiadiazolo(3,2-α)pyrimidine nucleus is associated with diverse pharmacodynamic and chemotherapeutic activities [18,19], including antimicrobial [19,20,21] and antitumor activities [18,20], herbicidal, antifungal, neuramidase inhibitors. 1,3,4-thiadiazolo(3,2-α)pyrimidines have been used as key building blocks for the preparation of a variety of novel bioactive agents [22], therefore, we thought it worthwhile to explore this coupled heterocyclic system for evaluation of anticancer activity. The designing protocol for a targeted compound is as shown in Figure 1.

The conventional multistep methods for the preparation of complex molecules involve large synthetic operations, including extraction and purification processes for each individual step, that lead to synthetic inefficiency and the generation of large amounts of waste. Therefore, designing multicomponent reactions (MCRs) in one pot and the creation of several bonds in a single operation are the major challenges for modern organic chemistry.

The synthesis of heterocycles using toxic and hazardous chemicals which cause pollution has given birth to “Green Chemistry,” coined by Paul Anastas in 1998 [23]. Ultrasound-promoted synthesis is one of the green methods of synthesis which we have used in our present research work in order to prevent pollution. Ultrasound-promoted synthesis has various advantages over conventional synthetic techniques such as highly accelerated reaction rate, reasonably good yields, simple open systems, very low amount of solvents required, eco friendly method, clean heating system, neat and clean synthetic protocol, cheaper reagents and less extreme physical conditions, control on reaction parameters, milder reaction conditions.

The existing synthetic methodologies for (1,3,4)thiadiazolo(3,2-α)pyrimidine nucleus in a modular fashion are not straightforward and the synthetic routes involve multiple steps. For example, 1,3,4-thiadiazolo(3,2-α)pyrimidine-7-sulfonamide derivatives were synthesized from 5-aminol-3,4-thiadiazole-2-sulfonamide via a two steps approach [24]. Salimov et al. [25] prepared 2-bromo-7-methyl-5-oxo-5*H*-1,3,4-thiadiazolo(3,2-α)pyrimidine by two steps involving the addition of 2-aminothiadiazole derivatives to ethyl acetoacetate, tandem hydrolysis of the ester to the acid, and cyclization to give the ring-fused thiadiazolo(3,2-α)pyrimidines in PPA. Most of these are multistep protocols, which suffer from generation of by-products, low yields, and use of metal-containing reagents. Therefore, it is quite significant to develop the direct, efficient, and green alternative approaches to get the functionalized thiadiazolo(3,2-α)pyrimidine derivatives from viewpoint of green chemistry. Herein we reported a new, simple protocol for an environment friendly, rapid and convenient synthesis, and antitumor activity against MCF-7, K562, HeLa, PC-3 cancer cell lines, of novel 5-amino-2-(4-chlorophenyl)-7-substituted phenyl-8,8a-dihydro-7*H*-(1,3,4)thiadiazolo(3,2-α)pyrimidine-6-carbonitrile derivatives obtained in excellent yield through a one-pot three component condensation reaction of 5-(4-chlorophenyl)-1,3,4-thiadiazol-2 amine, aromatic aldehyde and malononitrile using sodium hydroxide as catalyst in ultrasound. Moreover, docking studies using thymidylate synthase (TS) enzyme are presented in this paper as well.

## 2. Result and Discussion

### 2.1. Chemistry

**Scheme 1:**
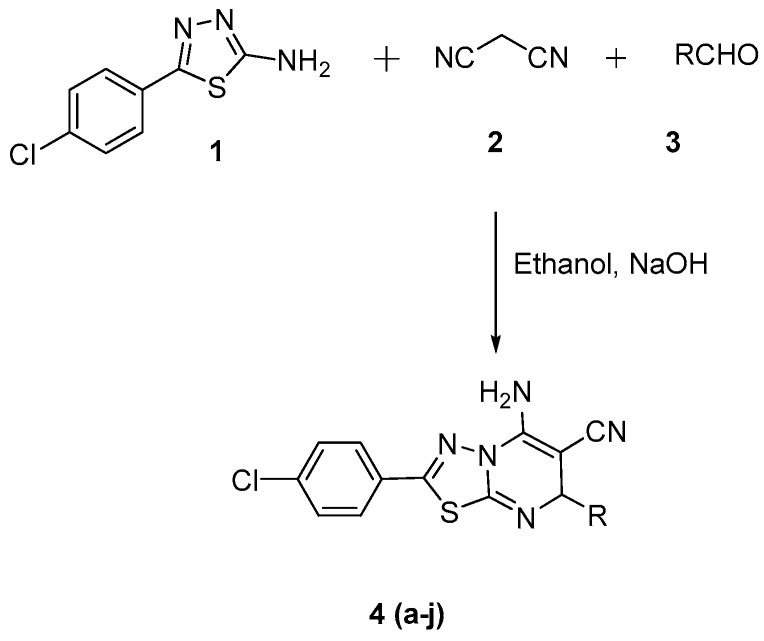
One-Pot, three component synthesis of novel 5-amino-2-(4-chlorophenyl)-7-substituted phenyl-8,8a-dihydro-7*H*-(1,3,4)thiadiazolo(3,2-α)pyrimidine-6-carbonitrile derivatives.

Herein we report the one-pot synthesis of novel 5-amino-2-(4-chlorophenyl)-7-substituted phenyl-8,8a-dihydro-7*H*-(1,3,4)thiadiazolo(3,2-α)pyrimidine-6-carbonitrile derivatives from three component reactions of an 5-(4-chlorophenyl)-1,3,4-thiadiazol-2 amine (**1**), aromatic aldehydes (**3**) and malononitrile (**2**) in presence of NaOH under reflux and ultrasonic irradiation as shown in Scheme 1. To determine the optimal reaction conditions, the one pot reactions between 5-(4-chlorophenyl)-1,3,4-thiadiazol-2 amine (**1**), suitable aldehyde (**3**), malononitrile (**2**) were carried out using different solvents in the presence of NaOH as a catalyst at different mole percentage as shown in Table 1, the desired product was not formed when H_2_O was chosen as solvent and instead acetonitrile, methanol and dimethylformaide was chosen as solvent. The desired product was formed in low yield under reflux and ultrasonic irradiation as shown in Table 1. The optimization of reaction conditions for 1,3,4-thiadiazolo(3,2-α)pyrimidine skeleton is as shown in Table 2. All the synthesized compounds were characterized by ^1^H-NMR, ^13^C-NMR, mass spectroscopy and IR.

### 2.2. In Vitro Anticancer Activity

The target compounds (**4a**–**j**) were evaluated for their anticancer activity against MCF-7, K562, HeLa and PC-3 cancer cell lines. The GI_50_ values (concentration required to Growth inhibition of 50%) for the synthesized compounds were determined using SRB assays method. The anticancer evaluation results and GI_50_ values were listed in Table 3 and the well-known anticancer drug 5-FU was used as positive control.

The results indicated that the compounds, **4e**, **4f**, **4g** and **4i** exhibited significant cell growth inhibition compared to reference standard 5-fluorouracil against MCF-7, K562, HeLa and PC-3 cancer cell lines. From the anticancer activity results, it was observed that compound **4i**, which has substituent 3-hydroxy-4-methoxyphenyl, is found to have the highest GI_50_ values of 32.7 μM, 34.3 μM, 55.3 μM, 28.9 μM for MCF-7, HeLa, K562 and PC-3 cancer cell lines respectively. Compound **4g**, which has 3,4-dimethoxyphenyl, is found to have GI_50_ values of 34.8 μM, 54.3 μM, 47.9 μM, 25.3 μM for MCF-7, K562, HeLa, and PC-3 cancer cell lines respectively. All the synthesized compounds were less active towards PC-3 in comparison to standard drug 5-FU.

Structural activity relationship (SAR) studies for these compounds demonstrated that electron withdrawing groups such as fluoro (**4d**), chloro (**4a**, **4b**, **4c**) exhibited less activity compared to electron donating, polar groups. The anticancer activity for derivatives bearing an electron withdrawing group such as chloro group at ortho position (**4b**) (43.8 μM, 57.1 μM, 54.3 μM, 37.9 μM for MCF-7, K-562, HeLa and PC-3 cancer cell lines respectively) exhibited more activity than compound **4c** (chloro group at meta position) (55.0 μM, 60.1 μM, 55.7 μM, 38.4 μM for MCF-7, K-562, HeLa and PC-3 cancer cell lines respectively) and compound **4a** (chloro group at para position) (88.5 μM, 47.9 μM, 56.2 μM, 38.9 μM for MCF-7, K-562, HeLa and PC-3 cancer cell lines respectively). Replacement of the phenyl group in the parent compound by furan ring in **4j** has shown decreased activity in comparison to the standard drug 5-FU (82.5 μM, >100 μM, 60.9 μM, 55.30 μM, for MCF-7, K-562, HeLa and PC-3 cancer cell lines, respectively). From SAR it can be considered that compounds containing electron donating, polar groups such as **4e**, **4f**, **4g**, **4i** have good anticancer activity in comparison to electron withdrawing groups such as **4a**, **4b**, **4c**, **4d**. It is also clear that the replacement of the phenyl ring with the furan ring decreases anticancer activity.

**4i** and **4g** are equipotent with the clinically used anticancer drug 5-FU against MCF-7, HeLa cell lines, hence these compounds can be developed as anticancer agents in the future. Compound **4a** is equipotent with the clinically used anticancer drug 5-FU against K-562.

### 2.3. Molecular Docking

**Figure 2:**
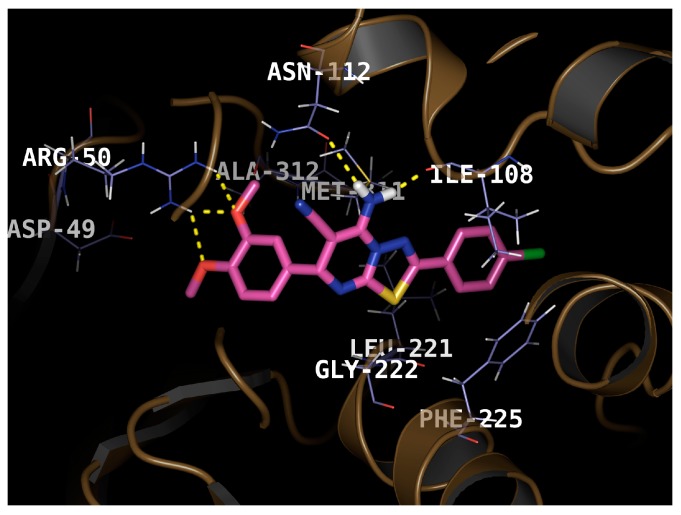
Docking pose of compound **4g** in the active site of thymidylate synthase enzyme.

**Figure 3:**
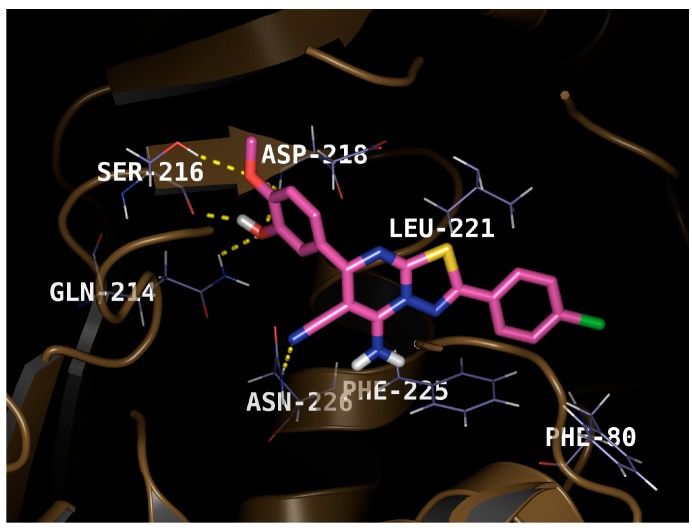
Docking pose of compound **4i** in the active site of thymidylate synthase enzyme.

5-Fluorouracil derivatives and their structurally related compounds like pemetrexed, Capecitibine, Raltitrexed are well known to inhibit thymidylate synthase. We decided to carry out a molecular docking study on our newly synthesized 1,3,4-thiadiazolo(3,2-α)pyrimidine skeleton derivatives into the binding site of thymidylate synthase (PDB ID: 1JU6) using 5-FU as a reference for docking results. The docking results indicated that compounds were held in the active pocket by combination of various hydrogen and hydrophobic interactions with TS. The docking results revealed that the highest binding compound to TS was **4i** with a G-Score of −7.17. The compound **4g** shows hydrogen bonding with ASN 112, ILE 108 and ARG 50 with a G-Score of −5.59 as shown in Figure 2. Similarly, the compound **4i** shows hydrogen bonding with GLN 214, SER 214 and ASN 226 with a highest G-Score of −7.17 as shown in Figure 3. The compounds **4b** (−5.59), **4c** (−5.24), **4d** (−5.14), **4f** (−5.14), **4h** (−5.38) and **4j** (−4.98), all showed G-Score greater than 5-FU (−4.75).

On the basis of the anticancer activity and docking results, it was found that compounds **4g**, **4h** and **4i** had potential to inhibit thymidylate synthase. G-Score of all the synthesized derivatives is as shown in Table 4.

### 2.4. In Silico ADME Prediction

The prediction of the ADME parameters prior to the experimental studies is one of the most important aspects of drug discovery and development of the drug molecule. ADME studies have always played a critical role in helping to optimize the pharmacokinetic properties of new drugs, thereby increasing their success rate. The analysis of Lipinski’s rule of five was performed to indicate whether a chemical compound could be an orally active drug in humans. It was observed that the compounds exhibited a good % absorption (% ABS) ranging from 87.50% to 100% (Table 5).

The results of the prediction of ADME properties are depicted in Table 5. All synthesized compounds **4a**–**j** had good absorption and were found to be nontoxic.

## 3. Materials and Methods

### 3.1. General

All the reactions were performed in oven-dried glasswares. All reagents and solvents were used as obtained from the supplier or recrystallized/redistilled unless otherwise noted. The ultrasound sonicator (Sonics Vibra-cell, Modelno. VCX 500, Newtown, CT, USA) equipped with solid synthetic probe, 13 mm in tip diameter, operating at 20 kHz with a maximum power output of 500 W, was used for synthesis of final title compounds. The purity of the synthesized compounds was monitored by ascending thin layer chromatography (TLC) on silica gel-G (Merck, Darmstadt, Germany) coated aluminum plates, visualized by iodine vapor and melting points were determined in open capillary tubes. Infrared (IR) spectra were recorded on a PS 4000 FTIR (JASCO, Tokyo, Japan) using KBr pellets. Elemental analyses (C, H, and N) were done with a FLASHEA 112 Shimadzu’ analyzer (Mumbai, Maharashtra, India) and all analyses were consistent (within 0.4%) with theoretical values. The ^1^H-NMR and ^13^C-NMR spectra of synthesized compounds were recorded on Bruker Avance II 400 NMR Spectrometer (Billerica, MA, USA) at 400 MHz Frequency in deuterated DMSO and CDCl_3_ and using TMS as internal standard (chemical shift δ in ppm). Mass spectra of some compounds were scanned on FTMS + p ESI full mass (100.00–1500.00).

### 3.2. General Procedure for the Synthesis of 5-Amino-2-(4-chlorophenyl)-7-Substituted Phenyl-8,8a-dihydro-7H-(1,3,4)thiadiazolo(3,2-α)pyrimidine-6-carbonitrile Derivatives

Method A: A 25 mL round bottom flask was charged with a mixture of an 5-(4-chlorophenyl)-1,3,4-thiadiazol-2 amine (0.01 mol) (**1**), malononitrile (0.01 mol) (**2**), suitable aldehyde (0.01 mol) (**3**) in ethanol (10–12 mL) and the catalyst NaOH (20% mmol) and the reaction mixture was refluxed . After completion of the reaction (monitored by TLC), the mixture was poured into ice cold water. The product obtained, was filtered and dried. The corresponding product was obtained in high purity after recrystallization of the crude product from ethanol. The authenticity of compounds was established by ^1^H-NMR, ^13^C-NMR, IR and HRMS.

Method B: A 25 mL a beaker was charged with a mixture of an 5-(4-chlorophenyl)-1,3,4-thiadiazol-2 amine (0.01 mol) (**1**), malononitrile (0.01 mol) (**2**), suitable aldehyde (0.01 mol) (**3**) in ethanol (10–12 mL) and the catalyst NaOH (20% mmol) and the reaction mixture was kept inside an Ultrasonicator acoustic chamber at 80 °C at 20%. After completion of the reaction (monitored by TLC), the mixture was poured into ice cold water. The product obtained, was filtered and dried. The corresponding product was obtained in high purity after recrystallization of the crude product from ethanol. The authenticity of compounds was established by ^1^H-NMR, ^13^C-NMR, IR and HRMS.

*5-Amino-2,7-bis(4-chlorophenyl)-7H-(1,3,4)thiadiazolo(3,2-α)pyrimidine-6-carbonitrile* **4a**. M.P: 237–240 °C. R*f* value: 0.28. IR (KBr) υmax cm^−1^: 3400 (C-NH_2_), 3100 (Aromatic C-H stretching), 1623 (C=N), 740.55 (C-Cl of aromatic rings), ^1^H-NMR δ ppm: 10.00 (s, 2H, NH_2_), 8.00–7.02 (m, 8H two aromatic rings), 3.35 (s, 1H, ArC, of pyrimidine ring), ^13^C-NMR δ: 172.11 (C), 158.31 (C), 143.21 (C), 139.50 (C), 136.51 (C), 131.00 (C), 130.52 (CH), 129.87 (CH), 128.53 (CH), 128.23 (C), 127.93 (CH), 118.27 (C), 60.22 (C), 54.53 (C), *m*/*z* 399 (100.0%), 401.01 (68.9%), 400.01 (22.7%) Molecular Formula: C_18_H_11_Cl_2_N_5_S. Elemental Analysis: Calculated: (C, H, Cl, N, S) 54.01, 2.77, 17.71, 17.50, 8.01 Found: 55.04, 2.74, 17.68, 17.51, 8.00.

*5-Amino-7-(2-chlorophenyl)-2-(4-chlorophenyl)7H-(1,3,4)thiadiazolo(3,2-α)pyrimidine-6-carbonitrile* **4b**. M.P: 240–242 °C. R*f* value: 0.24. IR (KBr) υmax cm^−1^: 3400 (C-NH_2_), 3000 (Aromatic C-H stretching), 1623 (C=N), 740.55 (C-Cl of aromatic rings). 1H-NMR δ ppm: 9.98 (s, 2H, NH_2_), 8.00–7.09 (m, 8H two aromatic rings), 3.35 (s, 1H, ArC, of pyrimidine ring), ^13^C-NMR δ ppm: 170.24 (C), 158.37 (C), 143.77 (C), 138.10 (C), 134.26 (C), 129.52 (CH), 129.00 (CH), 128.51 (CH), 128.03 (C), 127.99 (CH), 126.11 (CH), 117.59 (C), 60.00 (C), 55.02 (C), *m*/*z* 399 (100.0%), 401.01 (68.9%), 400.01 (22.7%) Molecular Formula: C_18_H_11_C_l2_N_5_S. Elemental Analysis: Calculated: (C, H, Cl, N, S) 54.01, 2.77, 17.71, 17.50, 8.01 Found: 55.02, 2.76, 17.70, 17.52, 8.02.

*5-Amino-7-(3-chlorophenyl)-2-(4-chlorophenyl)7H-(1,3,4)thiadiazolo(3,2-α)pyrimidine-6-carbonitrile* **4c**. M.P: 235–238 °C. R*f* value: 0.20. IR (KBr) υmax cm^−1^: 3400 (C-NH_2_), 3100 (Aromatic C-H stretching), 162.3 (C=N), 740.55 (C-Cl of aromatic rings), 1H-NMR δ ppm: 10.01 (s, 2H, NH_2_), 8.00–7.05 (m, 8H two aromatic rings), 3.32 (s, 1H, ArC, of pyrimidine ring), ^13^C-NMR δ ppm: 172.21 (C), 158.20 (C), 155.00 (C), 142.50 (C), 137.69 (C), 136.17 (C), 134.09 (C), 130.03 (CH), 129.99 (CH), 129.02 (CH), 128.93 (CH), 128.37 (CH), 125.59 (CH), 123.95 (CH), 119.11 (CH), 117.57 (C), 59.97 (C), 52.00 (C), *m*/*z* 399 (100.0%), 401.01 (68.9%), 400.01 (22.7%), Molecular Formula: C_18_H_11_C_l2_N_5_S. Elemental Analysis: Calculated: (C, H, Cl, N, S) 54.01, 2.77, 17.71, 17.50, 8.01 Found: 55.01, 2.77, 17.67, 17.49, 8.03.

*5-Amino-2-(4-chlorophenyl)-7-(4-fluorophenyl)-7H-(1,3,4)thiadiazolo(3,2-α)pyrimidine-6-carbonitrile* **4d**. M.P: 239–240 °C. R*f* value: 0.37. IR (KBr) υmax cm^−1^: 3400 (C-NH_2_), 3000 (Aromatic C-H stretching), 1623 (C=N), 740.55 (C-Cl of aromatic ring), 1053 (C-F of aromatic rings), ^1^H-NMR δ ppm: 9.79 (s, 2H, NH_2_), 8.02–7.06 (m, 8H two aromatic rings), 3.33 (s, 1H, ArC, of pyrimidine ring), ^13^C-NMR δ ppm: 172.22 (C), 159.07 (C), 158.31 (C), 143.77 (C), 136.62 (C), 136.99 (C), 130.67 (CH), 129.52 (CH), 128.99 (C), 128.77 (CH), 117.54 (C), 115.34 (CH), 60.07 (C), 52.97 (C), *m*/*z* 383.04 (100%), 385.04 (37%), 384.04 (22.7%), Molecular Formula: C_18_H_11_ClFN_5_S. Elemental Analysis: Calculated: (C, H, Cl, F, N, S) 56.33, 2.89, 9.24, 4.95, 18.25, 8.35 Found: 56.37, 2.90, 9.20, 4.91, 18.23, 8.36.

*5-Amino-2-(4-chlorophenyl)-7-(4-methoxyphenyl)-7H-(1,3,4)thiadiazolo(3,2-α)pyrimidine-6-carbonitrile* **4e**. M.P: 210–212 °C. R*f* value: 0.36. IR (KBr) υmax cm^−1^: 3400 (C-NH_2_); 3000 (Aromatic C-H stretching), 1623 (C=N), 1055 (C-OCH_3_), 740.55 (C-Cl of aromatic ring), ^1^H-NMR δ ppm: 9.99 (s, 2H, NH_2_), 8.02–6.79 (m, 8H two aromatic rings), 3.36 (s, 1H, ArC, of pyrimidine ring), 3.56 (s, 3H, OCH_3_), ^13^C-NMR δ ppm: 172.26 (C), 158.99 (C), 157.51 (C), 143.70 (C), 136.99 (C), 133.04 (C), 130.07 (CH), 129.55 (CH), 128.52 (CH), 128.00 (C), 117.37 (C), 114.26 (CH), 60.00 (C), 55.99 (CH_3_), 53.00 (C), *m*/*z* 395.06 (100%), 397.06 (37.0%), 396.06 (23.8%), Molecular Formula: C_19_H_14_ClN_5_OS. Elemental Analysis: Calculated: (C, H, Cl, N, O, S) 57.65, 3.56, 8.96, 17.69, 4.04, 8.10 Found: 57.68, 3.59, 8.93, 17.67, 4.03, 8.11.

*5-Amino-2-(4-chlorophenyl)-7-(3,4,5-trimethoxyphenyl)-7H-(1,3,4)thiadiazolo(3,2-α)pyrimidine-6-carbonitrile* **4f**. M.P: 220–222 °C. R*f* value: 0.26. IR (KBr) υmax cm^−1^: 3400 (C-NH_2_), 3100 (Aromatic C-H stretching), 1623 (C=N), 1059 (C-OCH_3_), 740.55 (C-Cl of aromatic ring), ^1^H-NMR δ ppm: 10.00 (s, 2H, NH_2_), 8.02–6.79 (m, 6H two aromatic rings), 3.36 (s, 1H, ArC, of pyrimidine ring), 3.56 (s, 9H, OCH_3_), ^13^C-NMR δ ppm: 172.21 (C), 158.89 (C), 152.81 (C), 143.32 (C), 136.52 (C), 136.00 (C), 135.01 (C), 129.51 (CH), 128.52 (CH), 128.77 (C), 117.51 (C), 106.50 (CH), 61.00 (CH_3_), 60.52 (C), 56.00 (CH_3_), 53.00 (C), *m*/*z* 455.08 (100%), 457.08 (37.1%), 456.09 (23.7%), Molecular Formula: C_21_H_18_ClN_5_O_3_S. Elemental Analysis: Calculated: (C, H, Cl, N, O, S) 55.32, 3.98, 7.78, 15.36, 10.53, 7.03 Found: 55.37, 3.99, 7.77, 15.33, 10.52, 7.00.

*5-Amino-2-(4-chlorophenyl)-7-(3,4-dimethoxyphenyl)-7H-(1,3,4)thiadiazolo(3,2-α)pyrimidine-6-carbonitrile* **4g**. M.P: 225–228 °C. R*f* value: 0.50. IR (KBr) υmax cm^−1^: 3400 (C-NH_2_), 3100 (Aromatic C-H stretching), 1623 (C=N), 1623 (C=N), 1052 (C-OCH_3_), 740.55 (C-Cl of aromatic ring), ^1^H-NMR δ ppm: 10.02 (s, 2H, NH_2_), 8.02–6.79 (m, 7H two aromatic rings), 3.36 (s, 1H, ArC, of pyrimidine ring), 3.56 (s, 6H, OCH_3_), ^13^C-NMR δ ppm: 172.07 (C), 158.00 (C), 149.07 (C), 146.72 (C), 143.54 (C), 136.58 (C), 134.51 (C), 129.59 (CH), 128.55 (CH), 128.70 (C), 122.01 (C), 117.56 (C), 114.99 (CH), 112.53 (CH), 59.11 (C), 56.09 (CH_3_), 53.00 (C), *m*/*z* 425.07 (100.0%), 427.07 (37.0%), 426.07 (24.09%), Molecular Formula: C_20_H_16_ClN_5_O_2_S. Elemental Analysis: Calculated: (C, H, Cl, N, O, S) 56.40, 3.79, 8.32, 16.44, 7.51, 7.53 Found: 56.44, 3.82, 8.30, 16.00, 7.50, 7.52.

*5-Amino-2-(4-chlorophenyl)-7-phenyl-7H-(1,3,4)thiadiazolo(3,2-α)pyrimidine-6-carbonitrile* **4h**. M.P: 218–220 °C. R*f* value: 0.43. IR (KBr) υmax cm^−1^: 3400 (C-NH_2_), 3000 (Aromatic C-H stretching), 1623 (C=N), 740.55 (C-Cl of aromatic ring), ^1^H-NMR δ ppm: 10.00 (s, 2H, NH_2_), 8.02–7.27 (m, 9H two aromatic rings), 3.34 (s, 1H, ArC, of pyrimidine ring), ^13^C-NMR δ ppm: 172.12 (C), 158.37 (C), 143.77 (C), 141.00 (C), 136.50 (C), 129.55 (CH), 129.08 (CH), 128.61 (C), 128.52 (CH), 125.79 (CH), 125.60 (CH), 117.51 (C), 60.00 (C), 53.12 (C), *m*/*z* 365.05 (100.0%), 367.05 (37.0%), 366.05 (22.7%), Molecular Formula: C_18_H_12_ClN_5_S. Elemental Analysis: Calculated: (C, H, Cl, N, S) 59.09, 3.31, 9.69, 19.14, 8.76 Found: 59.13, 3.33, 9.67, 19.10, 8.75.

*5-Amino-2-(4-chlorophenyl)-7-(3-hydroxy-4-methoxyphenyl)-7H-(1,3,4)thiadiazolo(3,2-α)pyrimidine-6-carbonitrile* **4i**. M.P: 240–245 °C. R*f* value: 0.51. IR (KBr) υmax cm^−1^: 3400 (C-NH_2_), (C-OH) 3333, 3000 (Aromatic C-H stretching), 1623 (C=N), 1055 (C-OCH_3_), 740.55 (C-Cl of aromatic ring), ^1^H-NMR δ ppm: 10.00 (s, 2H, NH_2_), 8.00–6.57 (m, 9H two aromatic rings), 5.35 (s, 1H, OH), 3.56 (s, 3H, OCH_3_), 3.34 (s, 1H, ArC, of pyrimidine ring), ^13^C-NMR δ ppm: 172.11 (C), 158.29 (C), 147.17 (C), 147.00 (C), 143.77 (C), 136.69 (C), 134.91 (C), 129.54 (CH), 128.69 (CH), 128.56 (C), 122.63 (CH), 117.58 (C), 115.07 (CH), 112.62 (CH), 60.00 (C), 56.02 (CH_3_), 53.07 (C), *m*/*z* 411.06 (100.0%), 413.05 (36.4%), 412.06 (22.2%), Molecular Formula: C_19_H_14_ClN_5_O_2_S. Elemental Analysis: Calculated: (C, H, Cl, N, O, S) 55.41, 3.43, 8.61, 17.00, 7.77, 7.79 Found: 55.44, 3.44, 8.59, 17.00, 7.79, 7.80.

*5-Amino-2-(4-chlorophenyl)-7-(furan-2-yl)-7H-(1,3,4)thiadiazolo(3,2-α)pyrimidine-6-carbonitrile* **4j**. M.P: 200–210 °C. R*f* value: 0.49. IR (KBr) υmax cm^−1^: (C-NH_2_), 3000 (Aromatic C-H stretching), 1623 (C=N), 740.55 (C-Cl of aromatic ring), ^1^H-NMR δ ppm: 9.08 (s, 2H, NH_2_), 7.77–7.55 (m, 4H two aromatic rings), 7.52 d, 1H, furan ring), 6.40 (t, 1H, furan ring), 6.08 (d, 1H, furan ring), 3.71 (s, 1H, ArC, of pyrimidine ring), ^13^C-NMR δ ppm: 172.17 (C), 157.77 (C), 152.59 (C), 144.32 (C), 142.36 (CH), 136.71 (C), 129.75 (CH), 129.00 (CH), 128.70 (C), 117.29 (C), 111.00 (CH), 105.97 (CH), 59.60 (C), 54.37 (C), *m*/*z* 355.03 (100%), 357.03 (37.1%), 356.03 (20.5%), Molecular Formula: C_16_H_10_ClN_5_OS. Elemental Analysis: Calculated: (C, H, Cl, N, O, S) 54.01, 2.83, 9.96, 19.68, 4.56, 9.01 Found: 54.05, 2.85, 9.95, 19.67, 4.56, 9.00.

### 3.3. Anticancer Screening

Experimental procedure for SRB assay [26].

The cell lines were grown in RPMI 1640 medium containing 10% fetal bovine serum and 2 mM l-glutamine. For the present screening experiment, cells were inoculated into 96 well microtiter plates in 90 µL at 5000 cells per well. After cell inoculation, the microtiter plates were incubated at 37 °C, 5% CO_2_, 95% air and 100% relative humidity for 24 h prior to addition of experimental drugs. Experimental drugs were solubilized in an appropriate solvent to prepare stock of 10^−2^ concentration. At the time of experiment, four 10-fold serial dilutions were made using complete medium. Aliquots of 10 µL of these different drug dilutions were added to the appropriate micro-titer wells already containing 90 µL of medium, resulting in the required final drug concentrations.

After compound addition, plates were incubated at standard conditions for 48 h and the assay was terminated by the addition of cold TCA. Cells were fixed in situ by the gentle addition of 50 µL of cold 30% (*w*/*v*) TCA (final concentration, 10% TCA) and incubated for 60 min at 4 °C. The supernatant was discarded; the plates were washed five times with tap water and air dried. Sulforhodamine B (SRB) solution (50 µL) at 0.4% (*w*/*v*) in 1% acetic acid was added to each of the wells, and plates were incubated for 20 min at room temperature. After staining, unbound dye was recovered and the residual dye was removed by washing five times with 1% acetic acid. The plates were air dried. Bound stain was subsequently eluted with 10 mM trizma base, and the absorbance was read on an Elisa plate reader at a wavelength of 540 nm with 690 nm reference wavelength.

Percent growth was calculated on a plate-by-plate basis for test wells relative to control wells. Percent Growth was expressed as the ratio of average absorbance of the test well to the average absorbance of the control wells × 100. Using the six absorbance measurements (time zero (Tz), control growth (C), and test growth in the presence of drug in the four concentration levels (Ti)), the percentage growth was calculated at each of the drug concentration levels. The dose response parameters were calculated for each test article. Growth inhibition of 50% (GI_50_) was calculated from ((Ti − Tz)/(C − Tz)) × 100 = 50, which is the drug concentration resulting in a 50% reduction in the net protein increase (as measured by SRB staining) in control cells during the drug incubation. The drug concentration resulting in total growth inhibition (TGI) was calculated from Ti = Tz.

Values were calculated for each of these three parameters if the level of activity was reached; however, if the effect was not reached or was exceeded, the values for that parameter were expressed as greater or less than the maximum or minimum concentration tested.

### 3.4. Molecular Docking

Molecular Docking Studies were performed in Maestro 9.1 using Glide v6.8 (Schrodinger, LLC, New York, NY, USA). All compounds were built using Maestro build panel and optimized to lower energy conformers using Ligprep v3.5.9 (Schrodinger, LLC) The coordinates for thymidylate synthase enzyme were taken from RCSB Protein Data Bank and prepared for docking using ‘protein preparation wizard’ in Maestro v10.3. (Schrodinger, LLC) The bond orders and formal charges were added for heterogroups and hydrogens were added to all atoms in the structure. Side chains that are not close to the binding cavity and do not participate in salt bridges were neutralized and termini were capped by adding ACE and NMA residue. After preparation, the structure was refined to optimize the hydrogen bond network using OPLS_2005 force field. The minimization was terminated when the energy converged or the RMSD reached a maximum cutoff of 0.30 Å. The extra precision (XP) docking mode for all compounds was performed on generated grid of protein structure [27,28,29,30,31]. The final evaluation of ligand-protein binding was done with Glide score.

### 3.5. In Silico ADME Prediction

A computational study of synthesized compounds **4a**–**j** was performed for prediction of ADME properties. The absorption, distribution, metabolism and excretion (ADME) properties of all compounds were predicted using Qikprop v3.5 (Schrödinger LLC). In the present study, we have calculated the molecular volume (MV), molecular weight (MW), Predicted octanol-water partition coefficient (log Po/w), number of hydrogen bond acceptors (*n*-ON), number of hydrogen bonds donors (*n*-OHNH), Percentage human oral absorption (% ABS), Polar surface area (PSA). The above described properties help us in understanding the ADME properties of any drug/synthesized molecule. A molecule likely to be developed as an orally active drug candidate should show no more than one violation of the following four criteria: log Po/w (octanol-water partition coefficient) ≤ 5, molecular weight ≤ 500, number of hydrogen bond acceptors ≤ 10 and number of hydrogen bond donors ≤ 5 [32].

## 4. Conclusions

The present work reports green synthetic route leading to the formation of antitumor active 5-amino-2-(4-chlorophenyl)-7-substituted phenyl-8,8a-dihydro-7*H*-(1,3,4)thiadiazolo(3,2-α) pyrimidine-6-carbonitrile and gives an impression about a clue for the mode of action by performing molecular docking study. The present protocol is also extendable to a wide variety of substrates. The advantages of this protocol are the use of eco-friendly catalyst, short reaction time, easy work-up, ease of product isolation, and high yield. From the results of anticancer activity and docking study, it is observed that the 1,3,4-thiadiazolo(3,2-α)pyrimidine skeleton is essential for the anticancer activity. Compounds containing electron donating, polar groups such as **4e**, **4f**, **4g**, **4i** have good anticancer activity in comparison to electron withdrawing groups. Among all the synthesized derivatives, **4g** and **4i** were equipotent to standard drug 5-FU. Compound **4i**, which has substituent 3-hydroxy-4-methoxyphenyl, is found to have the highest GI_50_ value of 32.7 μM, 55.3 μM, 34.3 μM, 28.9 μM for MCF-7, K562, HeLa and PC-3 cancer cell lines respectively. Compound **4g**, which has 3,4-dimethoxyphenyl, is found to have GI_50_ values of 34.8 μM, 54.3 μM, 38.7 μM, 34.7 μM for MCF-7, K562, HeLa and PC-3 cancer cell lines respectively. Finally, the ADME study shows that the synthesized drugs showed good drug like properties and opens the gateway for further optimization of studied compounds.

## Figures and Tables

**Table 1:** Optimization of reaction conditions for novel 5-amino-2-(4-chlorophenyl)-7-substituted phenyl-8,8a-dihydro-7*H*-(1,3,4)thiadiazolo(3,2-α)pyrimidine-6-carbonitrile derivatives using various solvent and different mole percentage of NaOH.

Entry	Catalyst	Amount (% mol)	Solvent	Method A Conventional		Method B Ultrasound	
				Time (h)	Yield (%)	Time (h)	Yield (%)
1	No catalyst	-	EtOH	9	-	2	-
2	NaOH	30	EtOH	9	70	2	89
3	NaOH	20	EtOH	9	70	2	89
4	NaOH	20	H_2_O	9	-	2	-
5	NaOH	20	MeOH	11	55	2.30	60
6	NaOH	20	CH_3_CN	11	40	2.30	50
7	NaOH	20	DMF	12	40	2.30	55
8	NaOH	10	EtOH	13	60	2.45	65
9	NaOH	5	EtOH	15	50	3.30	60

**Table 2:** Optimization of reaction conditions for 1,3,4-thiadiazolo(3,2-α)pyrimidine skeleton.

Compound	R	Conventional		Ultrasound	
		Time (h)	Yield (%)	Time (h)	Yield (%)
**4a**	4-chlorophenyl	7	70	1	89
**4b**	2-chlorophenyl	7.30	68	1.30	85
**4c**	3-chlorophenyl	7.30	65	1.30	85
**4d**	4-flurophenyl	7	62	1.30	80
**4e**	4-methoxyphenyl	8	58	2	75
**4f**	3,4,5-methoxyphenyl	9	58	2	78
**4g**	3,4-methoxyphenyl	9	55	2	75
**4h**	Phenyl	7	60	1	80
**4i**	3-hydroxy-4 methoxyphenyl	9	60	2	82
**4j**	furan-2-yl	8	45	1.30	65

**Table 3:** In-vitro anticancer activity.

Compound	GI_50_ μM			
	MCF-7	K-562	HeLa	PC-3
**4a**	88.5	47.9	56.2	38.9
**4b**	43.8	57.1	54.3	37.9
**4c**	55.0	60.1	55.7	38.4
**4d**	80.6	>100	58.1	30.2
**4e**	38.9	54.2	43.8	26.7
**4f**	38.3	58.1	48.6	25.4
**4g**	34.8	54.3	47.9	25.3
**4h**	38.9	58.3	38.7	34.7
**4i**	32.7	55.3	34.3	28.9
**4j**	82.5	>100	60.9	55.3
5-FU	32.18	47.03	43.71	12.00

5-Flurouracil (5-FU) was the standard drug used. GI_50_ is the concentration exhibiting 50% inhibition of the growth as compared to the growth of control. MCF-7 Human breast cancer, K-562 Human Leukemia cancer, HeLa Human cervical cancer, PC-3 Human prostate cancer.

**Table 4:** G-Score of all the synthesized derivatives.

Compound	G-Score	Compound	G-Score
**4a**	−4.65	**4g**	−5.59
**4b**	−5.59	**4h**	−5.38
**4c**	−5.24	**4i**	−7.17
**4d**	−5.14	**4j**	−4.98
**4e**	−4.74	5-FU	−4.75
**4f**	−5.14		

**Table 5:** Absorption, distribution, metabolism and excretion (ADME) results of synthesized compounds.

Compound	MW ^a^	Percentage of Absorption (% ABS) ^b^	logP o/w ^c^ (–2.0 to 6.5)	*n*-ON ^d^ (<10)	*n*-OHNH ^e^ (<5)	Polar Surface Area (PSA) ^f^ (7–200.0)	Number of Violations of Rule of 5 (<5)	Toxicity
**4a**	400	100	4.57	4	2	74.81	0	N
**4b**	400	100	4.42	4	2	74.87	0	N
**4c**	400	100	4.57	4	2	74.81	0	N
**4d**	383.8	100	4.30	4	2	74.81	0	N
**4e**	395.8	100	4.19	4.75	2	83.10	0	N
**4f**	455.9	100	4.47	6.25	2	95.95	0	N
**4g**	425.8	100	4.36	5.5	2	88.02	0	N
**4h**	365.8	100	4.01	4	2	74.81	0	N
**4i**	411.8	87.50	3.46	5.5	3	104.7	0	N
**4j**	355.8	94.19	3.12	4.5	2	83.51	0	N
5-FU	130.0	66.30	−0.89	3.5	2	87.56	0	N

^a^ Molecular weight of the molecule; ^b^ Percentage human oral absorption (% ABS) (>80% is high, <25% is poor); ^c^ Predicted octanol-water partition coefficient (logP o/w) (–2.0 to 6.5); ^d^ *n*-ON number of hydrogen bond acceptors <10; ^e^ *n*-OHNH number of hydrogen bonds donors <5; ^f^ Polar surface area (PSA) (7.0–200.0); N: non-toxic.
